# The Capsular Polysaccharides GXM and GXMGal from *Cryptococcus neoformans* Modulate Macrophages Infected with *Leishmania major*

**DOI:** 10.3390/microorganisms13102272

**Published:** 2025-09-28

**Authors:** Idália Maria Ferreira-dos-Santos, Elias Barbosa da Silva-Junior, Afonso Santine M. M. Velez, Leonardo Freire-de-Lima, Alexandre Morrot, Marco Edilson Freire de Lima, Gustavo José Makhoul, Joyce Cristina Guimarães-de-Oliveira, Israel Diniz-Lima, Luciana Polaco Covre, Renata Quintanilha dos Santos, Fernanda de Paula Pepino, Letícia Seabra Abrantes, Lucia Helena Pinto-da-Silva, José Osvaldo Previato, Lucia Mendonça-Previato, Celio Geraldo Freire-de-Lima, Debora Decote-Ricardo

**Affiliations:** 1 Instituto de Biofísica Carlos Chagas Filho, Universidade Federal do Rio de Janeiro, Rio de Janeiro 21941-902, Brazil; idaliamariaa@gmail.com (I.M.F.-d.-S.); eliasbarbosa@biof.ufrj.br (E.B.d.S.-J.); leolima@biof.ufrj.br (L.F.-d.-L.); gustavoj.makhoul@gmail.com (G.J.M.); joycristina4@biof.ufrj.br (J.C.G.-d.-O.); israel@biof.ufrj.br (I.D.-L.); l.covre@biof.ufrj.br (L.P.C.); previato@biof.ufrj.br (J.O.P.); luciamp@biof.ufrj.br (L.M.-P.); 2 Instituto de Química, Universidade Federal Rural do Rio de Janeiro, Seropédica 23897-000, Brazil; afonsosv30@gmail.com (A.S.M.M.V.); marcoedilson@gmail.com (M.E.F.d.L.); 3 Instituto Oswaldo Cruz, FIOCRUZ, Rio de Janeiro 21045-900, Brazil; alexandre.morrot@ioc.fiocruz.br; 4 Faculdade de Medicina, Universidade Federal do Rio de Janeiro, Rio de Janeiro 21941-902, Brazil; 5 Departamento de Doenças Infecciosas, Universidade Federal do Espírito Santo, Vitória 29047-100, Brazil; 6 Instituto de Veterinária, Universidade Federal Rural do Rio de Janeiro, Seropédica 23890-000, Brazil; renataquintanilha@ufrrj.br (R.Q.d.S.); fernandapepino@ufrrj.br (F.d.P.P.); leticiaseabra@gmail.com (L.S.A.); lpinto@ufrrj.br (L.H.P.-d.-S.)

**Keywords:** *Leishmania major*, immunomodulatory, capsular polysaccharides, macrophages, GXM, GXMGal, infection

## Abstract

*Leishmania* spp. are obligatory intracellular parasites that primarily infect macrophages. The macrophage immune response plays a pivotal role in determining the control or progression of infection. “M1-like” macrophages mediate parasite clearance through the production of nitric oxide, pro-inflammatory cytokines, and reactive oxygen species, whereas “M2-like” macrophages contribute to infection progression by exerting anti-inflammatory effects. The capsular polysaccharides Glucuronoxylomannan (GXM) and glucuronoxylomannogalactan (GXMGal) from *Cryptococcus neoformans* are capable of immunomodulating the macrophage response. GXM exhibits immunoregulatory activity, whereas GXMGal induces a pro-inflammatory response. Although the activity of these polysaccharides has been studied in cryptococcosis, their immunomodulatory potential in other infectious models remains largely unexplored. Here, we investigated the effects of GXM and GXMGal on *Leishmania major* infection in murine peritoneal macrophages. Murine peritoneal macrophages were infected with *L. major* and, 24 h post-infection, treated with 50 μg of either GXM or GXMGal. Our data revealed that GXM treatment enhanced *L. major* infection, while GXMGal treatment had no significant effect on the parasitic load in infected macrophages.

## 1. Introduction

Leishmaniases are a group of neglected tropical diseases caused by parasites of the genus *Leishmania* spp., which manifest in cutaneous, mucocutaneous, and visceral forms [1]. With a significant impact in Europe, Asia, Africa, and Latin America, leishmaniases pose a major public health challenge, accounting for approximately 1 million new cases and over 20,000 deaths annually [2]. The life cycle of the parasite begins with sand fly vectors of the Phlebotomus or Lutzomyia genus, where infected macrophages containing amastigotes, an intracellular parasitic form, are ingested during blood feeding on an infected host. Within the vector, amastigotes undergo biochemical and morphological differentiation into promastigotes, which are subsequently inoculated into a new host during subsequent blood feeding. Once inoculated into the host skin, an inflammatory immune response is triggered, involving the initial participation of phagocytic cells such as macrophages and neutrophils [3,4,5].

Macrophages play an important role in the *Leishmania* life cycle, serving as the primary host cells in which promastigotes differentiate into amastigotes, thereby enabling parasite survival and multiplication. The functional phenotype adopted by macrophages during infection is crucial in determining the outcome of host–parasite interaction. Pro-inflammatory “M1-like” macrophages mediate parasite elimination through the production of reactive oxygen species, nitric oxide (NO), pro-inflammatory cytokines such as TNF-α and IL-6, as well as cyclooxygenase-2 (COX-2). In addition, they exhibit robust antigen-presenting capacity through major histocompatibility complex (MHC) II molecules [6,7,8,9,10,11]. In contrast, anti-inflammatory “M2-like” macrophages favor parasite persistence by secreting immunosuppressive cytokines such as TGF-β and IL-10 [8,12,13]. *Leishmania* has evolved multiple strategies to subvert macrophage function and promote intracellular survival [14,15,16,17]. Additional factors may also favor *Leishmania* infection, such as increased formation of intracellular droplets, which serve as nutrient reservoirs for the parasite. These organelles are sites of prostaglandin (PG) E2 synthesis, playing an important role in infection susceptibility by inhibiting NO production and promoting IL-10 release [18,19,20,21,22,23].

The capsular polysaccharides of *Cryptococcus neoformans* play a crucial role in modulating macrophage activity [24,25]. The polysaccharide GXM, the major constituent of the fungal capsule, can immunosuppress macrophage response by inducing the production of IL-10, IL-8, and upregulating the expression of FcRγII receptor [26,27,28,29]. Additionally, GXM also promotes the accumulation of TGF-β within macrophages and increases Fas/FasL expression, thereby inducing cell death via apoptosis [30]. Structurally, GXM consists of a mannose-based trisaccharide unit with xylosidic and glucuronosidic substitutions and acetylation [25,31,32]. Another polysaccharide that comprises the polysaccharide capsule is GXMGal [24,25]. Structurally, it is a polymer composed of a central chain of galactose with rare galactofuranose residues and side chain substitutions of galactomannan, containing xylosidic and glucuronosidic substitutions in the residues of mannose and galactose [32,33,34,35]. In contrast to GXM, which exerts predominantly anti-inflammatory effects, GXMGal induces the secretion of pro-inflammatory cytokines, such as IL-6 and TNF-α [36,37,38].

Despite the scarcity of studies investigating these polysaccharides in different infectious models, GXM and GXMGal are considered promising tools for the development of new therapeutic strategies [39,40]. In this context, we investigated the modulatory effects of GXM and GXMGal on murine macrophages infected with *L. major*. Our results indicate that GXM enhances *L. major* infection in murine macrophages.

## 2. Materials and Methods

### 2.1. Ethics Statement

This research was conducted strictly adhering to the guidelines outlined in the Guide for the Care and Use of Laboratory Animals of the National Institutes of Health (USA). The experimental protocol was reviewed and approved by the Committee on the Ethics of Animal Experiments of the Health Science Center of the Federal University of Rio de Janeiro (CEUA-CCS, Permit Number: IBCCF 01200.001568/2013-87, protocol n^o^ 079/19, 11 November 2024). Every effort was made to minimize the animals’ suffering.

### 2.2. Animals and Parasites

The *Leishmania major* LV39 strain (MRHO/Sv/59/P) was isolated from the popliteal lymph node of infected BALB/c animals and maintained in vitro as proliferating promastigotes in Schneider’s medium (Sigma Chemical Co., St. Louis, MO, USA), supplemented with 2 mM L-glutamine, 2% sterile human urine, 10 μg/mL gentamicin, and 10% Fetal Bovine Serum (FBS) (Gibco, Spark, MD, USA). Promastigote cultures were maintained for up to three weeks without detectable loss of infectivity.

### 2.3. Acquisition of Purified Polysaccharides GXM and GXMGal from C. neoformans

The procedures for isolation and purification of GXM and GXMGal were carried out based on the protocol previously described [30,34]. Briefly, GXM was isolated from the wild-type strain of *C. neoformans* B3501, while GXMGal was obtained from the mutant strain CAP67. Polysaccharides were precipitated with cold ethanol, dissolved, dialyzed in distilled water, and subsequently lyophilized.

### 2.4. Macrophage Acquisition and Infection

Peritoneal macrophages were obtained by peritoneal lavage of BALB/c mice with chilled Dulbecco’s Modified Eagle Medium (DMEM) supplemented with L-glutamine, pyruvate, essential amino acids, and tryptophan. Subsequently, macrophages were seeded at 5 × 10^5^ cells per well in culture plates and incubated at 37 °C in 5% CO_2_ atmosphere for at least 4 h to allow adherence. Following this incubation time, peritoneal macrophages were infected with 5 × 10^6^ stationary-phase *Leishmania major* promastigotes (LV39 strain) in DMEM supplemented with 10% heat-inactivated Fetal Bovine Serum, L-glutamine, pyruvate, amino acids, and tryptophan.

### 2.5. Parasite Load Quantification

Intracellular amastigote quantification was performed following a 3-day culture of infected peritoneal macrophages on glass coverslips placed in 1 mL culture vessels (Corning), in the presence or absence of purified capsular polysaccharides (GXM or GXMGal). After the incubation period, coverslips were washed and stained using the May–Grunwald Giemsa protocol (Sigma-Aldrich, St. Louis, MO, USA) according to the manufacturer’s instructions. The numbers of intracellular amastigotes and infected macrophages were determined by light microscopy. Quantitative data are expressed as the number of amastigotes per 100 macrophages and as the percentage of infected cells. All data are presented as mean ± standard error (SE) from triplicate cultures.

For quantification of promastigote differentiation, infected macrophages were similarly cultured with or without purified capsular polysaccharides for 3 days. Subsequently, infected macrophage monolayers were washed and transferred to 0.5 mL of Schneider’s medium (Sigma-Aldrich), supplemented with 20% fetal bovine serum (FBS) and 2% human urine. The monolayers were then incubated at 26 °C for an additional 3 days. Motile extracellular promastigotes released into the supernatant were quantified by counting using a Neubauer chamber. Data are expressed as mean ± SE from triplicate cultures.

### 2.6. Treatment with GXM or GXMGal, Cytokines and Inhibitors 

Peritoneal macrophages were incubated overnight with 50 μg/mL of GXM or GXMGal, stimulated or not with 20 ng/mL of recombinant murine IFN-γ. To interfere with the cyclooxygenase pathway, peritoneal macrophages were treated with either 10 μg/mL of aspirin (Sigma-Aldrich) or 1 μM/mL of NS-398 (CaymanChem, Ann Arbor, MI, USA), or with an equivalent dose of vehicle (DMSO). For cytokine neutralization, monoclonal antibodies against IL-10, TGF-β or TNF-α were added to the cultures at a final concentration of 10 mg/mL. To inhibit nitric oxide (NO) production, macrophages were treated with 1 mM of L-NIL (CaymanChem, Ann Arbor, MI, USA), a selective inhibitor of inducible nitric oxide synthase.

### 2.7. Cytokine Assay

The concentrations of IL-10, TGF-β, and TNF-α in the supernatants of peritoneal macrophage cultures, infected or not with *L. major* were analyzed after 24 h of treatment or not with GXM or GXMGal, using a sandwich-type enzyme-linked immunosorbent assay (ELISA). Commercial ELISA development kits as IL-10 (Anti-Murine IL-10 Standard TMB ELISA Development kit (ThermoFischer^®^, Waltham, MA, USA), TGF-β (Anti-Murine TGF-β Standard TMB ELISA Development kit (ThermoFischer^®^, Waltham, MA, USA) and TNF-α (Anti-Murine TNF-alpha Standard TMB ELISA Development kit (ThermoFischer^®^, Waltham, MA, USA), were used according to the manufacturer’s instructions. The reading was performed in a spectrophotometer at 450 nm.

### 2.8. Nitric Oxide (NO) Assay

The NO production was analyzed by measuring nitrite levels in culture supernatants using the Griess reaction [41]. Briefly, aliquots of 50 μL of each sample was added to a flat-bottom 96-well plate and then incubated with 50 μL of Griess reagent (25 μL of 1% sulfanilamide solution in H_3_PO_4_ and 25 μL of 0.1% N-(1-naphthyl) ethylenediamine solution in H_2_O). Absorbance was measured at 540 nm using an automatic microplate reader.

### 2.9. Cell Viability 

Peritoneal macrophages were treated with GXM or GXMGal at an initial concentration of 300 μg/mL, followed by serial dilutions to obtain a concentration range. After 24 h of treatment at 37 °C in a 5% CO_2_ atmosphere, cell viability was assessed using the MTT assay. For this, 10 μL of MTT solution (5 mg/mL) was added to each well, and the plate was incubated for up to 1 h at 37 °C, in a 5% CO_2_ atmosphere, protected from light. The formation of intracellular formazan crystals was monitored every 20 min under an optical microscope. Upon confirmation of crystal formation, 100 μL of DMSO was added to each well to solubilize the crystals. The plate was then shaken for 15 min, protected from light, and absorbance was measured at 550 nm using an automated microplate reader.

### 2.10. Quantification of Lipid Droplets

Lipid droplets quantification was performed after 24 h of treatment or not with GXM or GXMGal. Wells containing coverslips were washed with 1× PBS at room temperature, and subsequently, infected cells were fixed with 3.7% paraformaldehyde for 30 min. After fixation, coverslips were washed twice with distilled water, followed by the addition of 200 μL of 0.1 M cacodylic acid and 200 μL of osmium tetroxide solution (1:1). Coverslips were incubated with this solution for 30 min. Then, wells containing coverslips were washed five times with distilled water and incubated with thiocarbohydrazide (TCH) for 5 min. After an additional five washes with distilled water, the cacodylic acid and osmium tetroxide solution (1:1) were added again for another 5 min. Coverslips were then washed five more times with distilled water, removed, fixed on a clean and dry slide with Entellan. Lipid droplets were quantified by optical microscope at 1000× magnification using immersion oil.

### 2.11. Prostaglandin-E_2_ (PGE_2_) Assay

The quantification of PGE_2_ was performed 24 h after treatment with GXM or GXMGal using a commercially specific PGE_2_ EIA kit (Anti-Murine PGE_2_ Standard TMB ELISA Development kit (ThermoFischer^®^, Waltham, MA, USA), according to the manufacturer’s instructions. 

### 2.12. Statistical Analysis

Statistical analyses were performed using GraphPad Prism version 9.5 (GraphPad Software, San Diego, CA, USA). For the cell viability assay, the Two-way ANOVA test was used, and for the other assays, differences between groups were determined using the One-way ANOVA test. Values of *p* ≤ 0.05 were considered statistically significant.

## 3. Results

### 3.1. GXM and GXMGal at Elevated Levels Do Not Induce Cytotoxicity in Murine Macrophages

**Figure 1:**
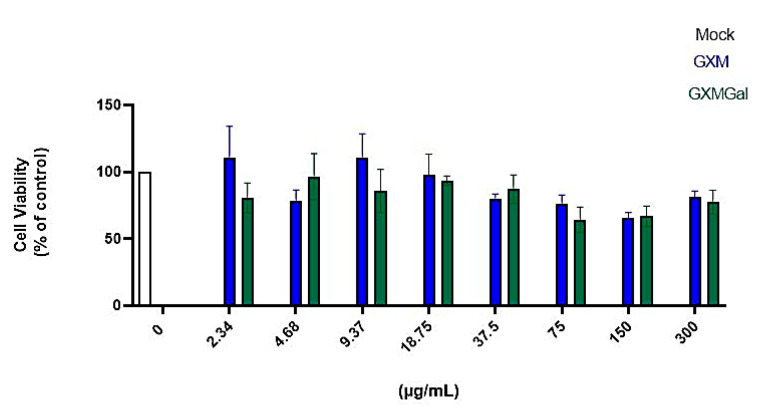
Cell viability assay. Peritoneal macrophages were treated for 24 h with different concentrations of GXM or GXMGal. Serial dilutions were performed to obtain concentrations as low as 2.34 μg/mL, as shown in the figure. After 24 h of treatment, cell viability was assessed using MTT assay. Absorbance was measured with a microplate reader at 550 nm. All cultures were performed in triplicate, and the data are representative of three independent experiments. Bars represent the mean ± SD. Statistical analysis was performed using One-Way ANOVA.

Initially, we began by evaluating which concentration of the capsular polysaccharides GXM and GXMGal would be suitable for treatment without causing cellular damage. We treated peritoneal macrophages with an initial concentration of 300 μg/mL, then performed a serial dilution to obtain different concentrations of the polysaccharides. After 24 h of treatment, we analyzed cell viability using the MTT assay (Figure 1). We found that concentrations ≤300 μg/mL of GXM and GXMGal did not affect cell viability, indicating that the tested concentrations are not toxic to macrophages. In this study, we defined the concentration of 50 μg/mL because it is a concentration already used in other studies by our group [30,38].

### 3.2. The Capsular Polysaccharide GXM Promotes L. major Infection in Murine Macrophages

**Figure 2:**
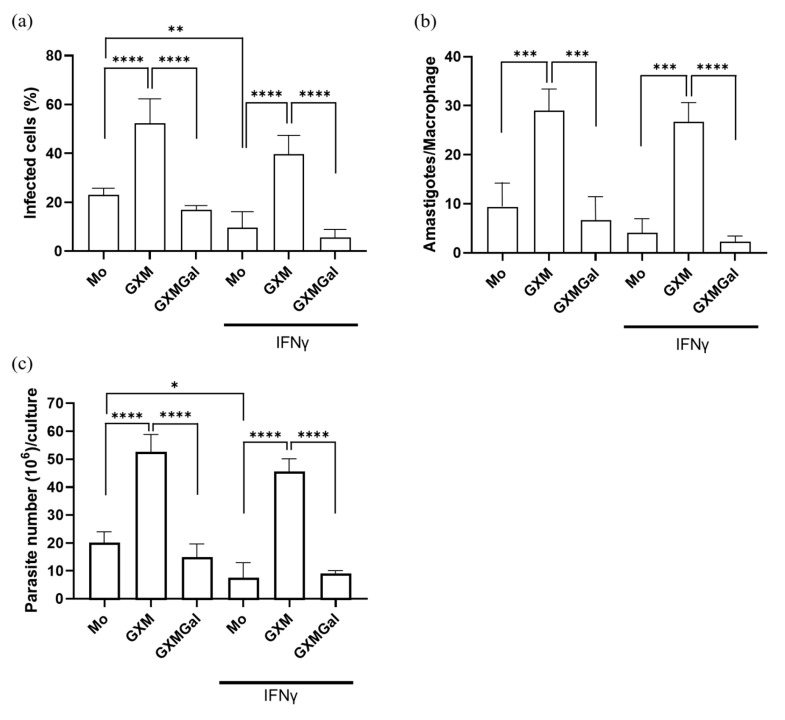
Quantification of the parasitic load of promastigotes and amastigotes in murine macrophages infected with *L. major*. (**a**–**c**) Murine peritoneal macrophages (5 × 10^5^/mL) were infected with promastigote forms of *L. major* (5 × 10^6^/mL) in 48-well plates at a 10:1 parasite/macrophage ratio. After 24 h of infection, the cultures were treated with 50 μg/mL of GXM or GXMGal in the presence or absence of IFN-γ (20 ng/mL) and incubated for 3 days. (**a**,**b**) On the third day, the cells were stained with a panoptic staining kit to determine the percentage of infected macrophages (**a**) and the number of amastigotes/macrophages (**b**). (**c**) On the third day, the cells were transferred to Schneider’s supplemented medium, and the plate was maintained in a B.O.D. incubator at 27 °C in a humid chamber for 12 days. The number of viable promastigotes was quantified by counting in a Neubauer chamber. (**a**–**c**) All experiments were performed in triplicate, and the data were representative of 3 independent experiments. Data is presented as mean ± SD. Statistical analysis was conducted using One-Way ANOVA. **** *p* ≤ 0.0001, *** *p* ≤ 0.001, ** *p* ≤ 0.01, * *p* ≤ 0.05.

To analyze whether the capsule polysaccharides from *Cryptococcus neoformans* influence the control of parasitic load, murine peritoneal macrophages were infected with promastigote forms of *L. major*. Twenty-four hours after infection, the cells were treated with 50 μg of GXM or GXMGal in the presence or absence of 20 ng of IFN-γ. After 3 days of treatment, we observed that the percentage of infected macrophages was lower in the GXMGal-treated group compared to the GXM (Figure 2a). Additionally, the quantification of intracellular amastigotes present in each macrophage revealed a significantly reduced number of amastigotes in macrophages treated with GXMGal compared to those treated with GXM (Figure 2b). During the 15 days of treatment, we quantified extracellular promastigotes of *L. major*. Again, we observed an increased number of promastigotes in GXM-treated cultures, whereas GXMGal treatment was associated with a reduced number of parasites (Figure 2c). Collectively, these results suggest that GXM promotes an intracellular microenvironment favorable for parasite growth, whereas GXMGal does not affect the macrophage’s ability to control parasite replication.

### 3.3. GXM Increases IL-10 and TGF-β Production, While GXMGal Increases TNF-α Production

**Figure 3:**
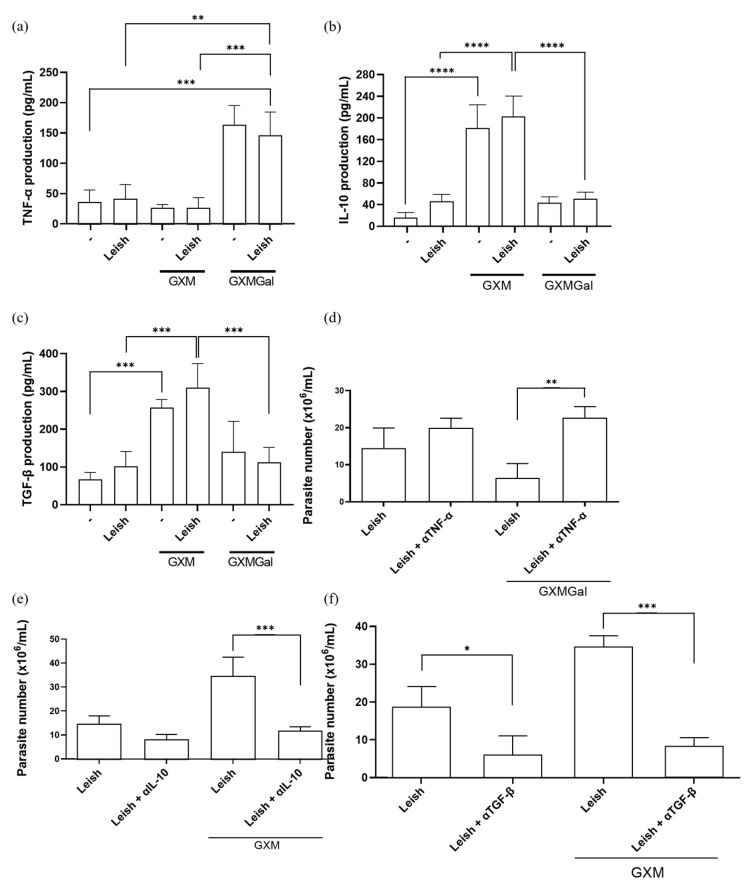
Measurement of cytokine production. (**a**–**c**) Peritoneal macrophages were infected with metacyclic promastigotes of *L. major* in 48-well plates at a multiplicity of infection (MOI) of 10:1. After 24 h of infection, cultures were treated with either GXM or GXMGal. Cytokine levels were quantified 24 h post-treatment using commercial ELISA kits, according to the manufacturer’s recommendation. Absorbance was measured at 450 nm. (**d**–**f**) Peritoneal macrophages infected with *L. major* and treated or not with GXM or GXMGa, were incubated in the presence or absence of neutralizing monoclonal antibodies targeting IL-10, TGF-β, or TNF-α, as well as an isotype control. After 3 days of incubation, cells were transferred to Schneider’s medium supplemented with 2% human urine and 10% FBS and subsequently maintained at 27 °C in a B.O.D. incubator within the humid chamber for 7 days. The number of viable promastigotes was determined using a Neubauer chamber. All cultures were performed in triplicate. Data were presented as mean ± SD. Statistical analysis was performed by One-Way Anova. **** *p* ≤ 0.0001, *** *p* ≤ 0.001, ** *p* ≤ 0.01, * *p* ≤ 0.05.

Given our hypothesis that both treatments modulate macrophage phenotype, we evaluated the production of key cytokines. As previously described, GXM is capable of immunosuppressive effects on macrophage activity by inducing IL-10 and TGF-β production, while GXMGal induces a pro-inflammatory profile by promoting the production of pro-inflammatory cytokines such as TNF-α [24,25,26,27,28,29,30,31,36,37,38]. Therefore, we decided to analyze the production of these cytokines in *L. major*-infected macrophages treated with either GXM or GXMGal. We observed that the increase in TNF-α production occurs in the groups treated with GXMGal, while IL-10 and TGF-β production occurs in the groups treated with GXM (Figure 3a–c). To determine whether this cytokine production influences infection outcome, we performed neutralization assays targeting TNF-α, TGF-β, and IL-10, followed by assessment of parasite burden. We observed that macrophages infected with *L. major* and treated with GXMGal were able to control the infection; however, TNF-α neutralization led to an increase in the number of parasites (Figure 3d). On the other hand, macrophages infected with *L. major* and treated with GXM failed to control the infection; however, neutralization of IL-10 and TGF-β significantly reduced the number of parasites (Figure 3e,f). These results reveal that parasite control is directly dependent on the cytokine profile induced by GXM and GXMGal.

### 3.4. GXMGal Induces Nitric Oxide Production in Peritoneal Macrophages Infected with L. major

**Figure 4:**
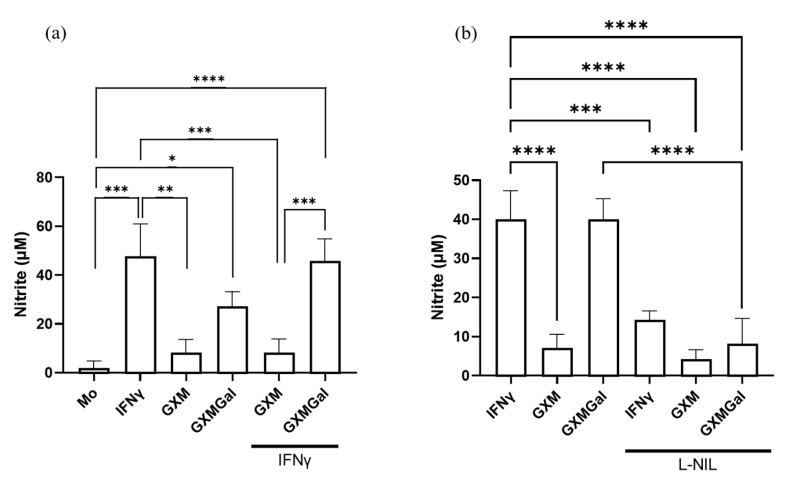
Measurement of nitrite. Peritoneal macrophages were infected with *L. major* promastigotes in 96-well plates at a multiplicity of infection (MOI) of 10:1. After 24 h of infection, cultures were treated with GXM or GXMGal in the presence or absence of IFN-γ. Nitrite production was assessed 6 h after treatment using the Griess method (**a**). The production of NO was inhibited using the inhibitor L-NIL (1 mM) (**b**). All conditions were performed in triplicate, and the data are presented as mean ± SD. Statistical analysis was performed by One-Way Anova. **** *p* ≤ 0.0001, *** *p* ≤ 0.001, ** *p* ≤ 0.01, * *p* ≤ 0.05.

Given the established role of nitric oxide production in mediating inflammatory responses and controlling parasite load, we sought to analyze whether treatment with polysaccharides could induce NO production [7]. For this purpose, infected peritoneal macrophages were treated with GXM or GXMGal, and NO levels were assessed 24 h later using the Griess reaction. We observed an increase in NO production only in cultures treated with GXMGal, both in the absence and presence of IFN-γ (Figure 4a). To confirm the specificity of this response, cultures were incubated with the selective NO inhibitor, L-NIL. Under these conditions, we observed a significant reduction in NO in cultures treated with GXMGal (Figure 4b). Collectively, our results demonstrate that GXMGal stimulates NO production in *L. major*-infected macrophages, suggesting a mechanism by which this polysaccharide contributes to parasite control.

### 3.5. GXM and GXMGal Increase PGE_2_ Production in Macrophages Infected with L. major

**Figure 5:**
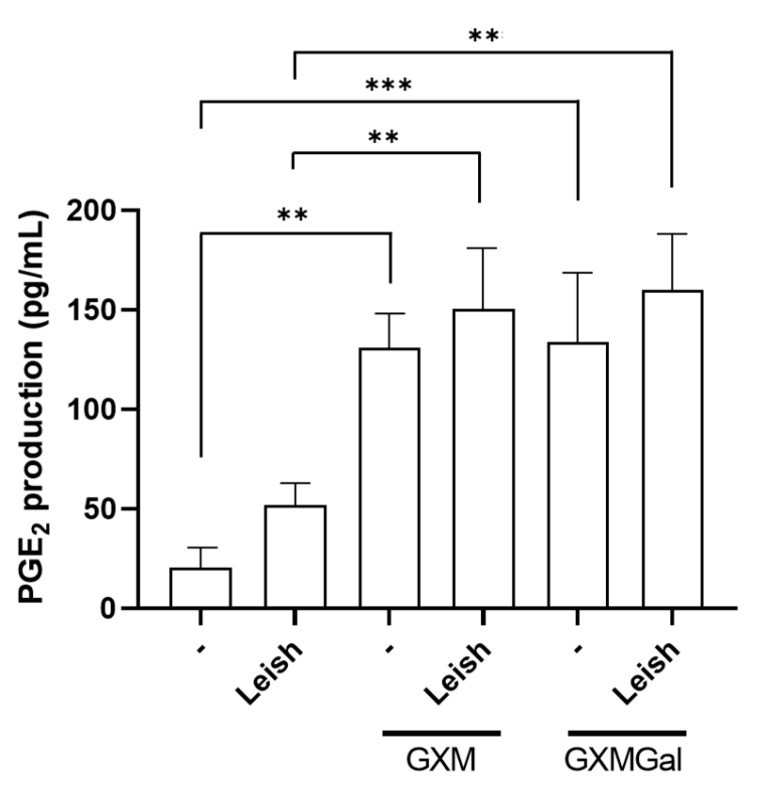
Measurement of PGE_2_ in peritoneal macrophages infected with *L. major* promastigotes. Murine peritoneal macrophages (2 × 10^5^/mL) were infected or not with *L. major* promastigote (2 × 10^6^/mL; MOI 10:1) in 48-well plates. After 24 h of infection, the cultures were treated with 50 µg/mL of GXM or GXMGal. PGE_2_ levels were measured 24 h post-treatment using a commercial ELISA kit, according to the manufacturer’s instructions. All cultures were performed in triplicate, and the data are representative of three independent experiments. Data represents the mean ± SD. Statistical analysis was conducted using One-Way ANOVA. *** *p* ≤ 0.001, ** *p* ≤ 0.01.

The production of PGE_2_ during *Leishmania* infection has been associated with the persistence of parasitic infection [42]. Thus, we analyzed whether the polysaccharides were inducing PGE_2_ synthesis in macrophages infected or not with *L. major*. Our results revealed that GXM and GXMGal are capable of inducing PGE_2_ production in macrophages, regardless of parasitic infection (Figure 5). Therefore, we suggest that the role of PGE_2_ in *Leishmania* infection may be influenced by pro-inflammatory or anti-inflammatory factors present in the microenvironment.

### 3.6. Macrophages Infected with L. major Regain the Ability to Control Parasitic Load After Inhibition of PGE_2_ Synthesis

**Figure 6:**
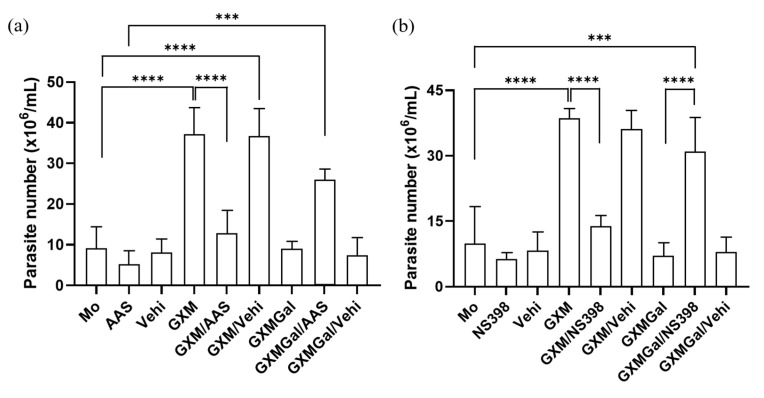
Inhibition of PGE_2_ synthesis modulates parasite burden in macrophages treated with GXM or GXMGal. Peritoneal macrophages, infected or not with *L. major*, were treated with (**a**) aspirin (10 mg/mL) or (**b**) NS-398 (1 mM) in the presence of GXM or GXMGal. After 24 h of treatment, cells were transferred to Schneider’s medium supplemented with 2% human urine and 10% FBS, and the plate was kept in a B.O.D. incubator at 27 °C in a humid chamber for 7 days. Promastigotes were quantified using a Neubauer chamber. All cultures were performed in triplicate, and bars represent mean ± SD. Statistical analysis was conducted by One-Way ANOVA. *** *p* ≤ 0.001; **** *p* ≤ 0.0001.

As we observed alterations in PGE_2_ synthesis induced by both GXM and GXMGal, we next investigated whether this lipid mediator plays a role in modulating parasite burden. For this purpose, we used two nonsteroidal anti-inflammatory drugs (NSAIDs), Aspirin (AAS) and NS-398. These drugs inhibit PGE_2_ production by targeting cyclooxygenase [43,44]. Our data demonstrated that both AAS and NS-398 significantly reduce the parasite burden in *L. major*-infected macrophages treated with GXM. Conversely, inhibition of PGE_2_ synthesis in *L. major-*infected macrophages treated with GXMGal led to an increase in parasite burden (Figure 6a,b). Thus, these findings demonstrate that PGE_2_ is involved in the regulation of parasite burden.

### 3.7. Both GXM and GXMGal Induced Lipid Droplet Formation in L. major-Infected Macrophages

**Figure 7:**
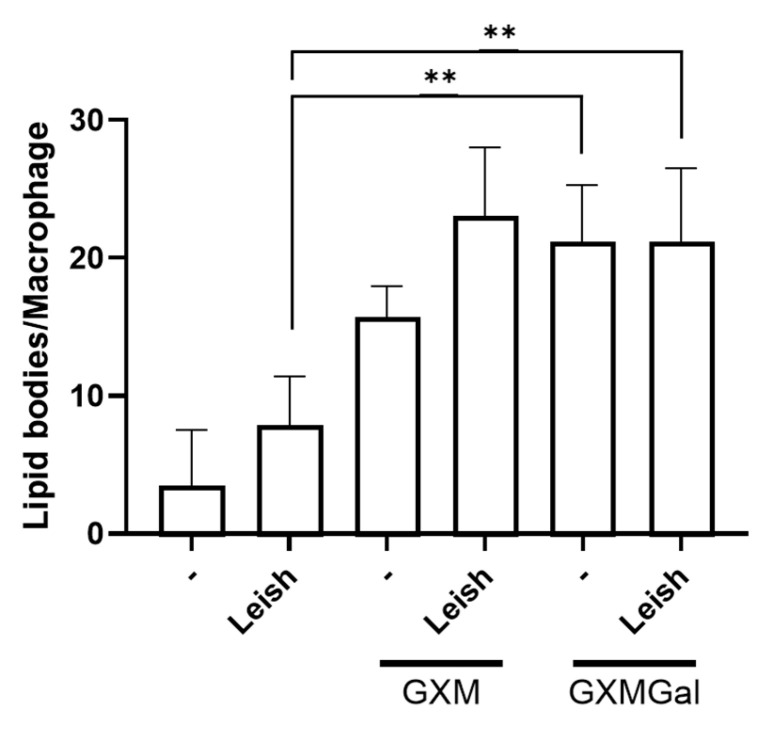
Lipid droplet quantification. Peritoneal macrophages were infected with *L. major* promastigotes (MOI 10:1). After 24 h of infection, cells were treated or not with GXM or GXMGal on coverslip. Following 24 h of treatment, cells were fixed with 3.7% paraformaldehyde for 30 min and subsequently stained with osmium tetroxide. Lipid droplets were quantified in 100 cells using optical microscope. All cultures were performed in triplicate, and the bars represent mean ± SD. Statistical analysis was performed by One-Way Anova. ** *p* ≤ 0.01.

Given the observed increase in PGE_2_ production in macrophages, whether infected or not with *Leishmania*, and treated with GXM or GXMGal, we next analyzed whether these polysaccharides were inducing lipid body formation. These organelles serve as sites for PGE_2_ synthesis via the action of cyclooxygenase enzyme [45]. To assess this, we performed osmium tetroxide staining and then quantified lipid bodies in 100 randomly selected macrophages. Our results show that both GXM and GXMGal significantly increase lipid body (LB) formation in macrophages, regardless of *L. major* infection status (Figure 7). These findings suggest that the previously observed increase in PGE_2_ occurs due to the enhanced LB formation. Furthermore, the increase in LBs induced by GXMGal does not necessarily translate into effective parasite control.

## 4. Discussion

The type of response developed by macrophages during *Leishmania* infection plays a crucial role in determining the control or progression of the infection [6,13]. M1 macrophages exhibit a pro-inflammatory response profile, characterized by the production of nitric oxide and TNF-α, whereas M2 macrophages display an anti-inflammatory response profile, creating a permissive microenvironment for parasite survival, as they are involved in functions promoting resolution of inflammation and tissue repair [6,8,10,13]. Macrophage polarization can be conditioned by various factors, such as cytokines present in the microenvironment, or modulated by the parasite itself, which has evolved mechanisms to subvert host cell responses and ensure its intracellular survival and replication [12,42,46]. The ability to subvert macrophage responses is also described for other types of pathogens, as seen in fungal infections [31,47,48].

The primary virulence factors of pathogenic fungi include the expression of glycoconjugates that compose their structural components [47,48,49,50,51,52]. In *Cryptococcus neoformans*, the capsule polysaccharides glucuronoxylomannan (GXM) and glucuronoxylomannogalactan (GXMGal) have immunomodulatory properties that may be relevant in the context of certain pathologies [39,40,53]. In addition, GXM from both *C. neoformans* and *C. gattii* can be deposited in various tissues of rats and mice [54,55]. Our group has been dedicated to understanding the immunopathology associated with the *Cryptococcus* genus and has demonstrated that both *C. neoformans* and *C. gattii* can modulate the host immune response by suppressing the Th1/Th17 antifungal response [38,56,57,58]. In this study, to further elucidate the immunomodulatory roles of GXM and GXMGal under different infection contexts and their potential contributions, we investigated the effects of these polysaccharides on macrophages infected with *L. major*.

We initially confirmed that polysaccharides were non-toxic to the cell cultures. Although previous studies have reported that GXM and GXMGal polysaccharides can induce cell death via apoptosis under certain conditions [30], the concentrations tested in this study did not compromise cell viability. Our group has routinely used a concentration of 50 μg/mL in prior studies; therefore, this concentration was maintained for the present study [30,38].

Upon analyzing the infection profile of *L. major* in macrophages treated with GXM or GXMGal, we observed a higher parasitic load of both amastigotes and promastigotes in the GXM-treated group, indicating impaired parasite clearance. In contrast, GXMGal treatment did not affect the parasitic load. These results led us to suggest that the immunosuppressive properties of GXM and the immunostimulatory properties of GXMGal might be conditioning different macrophage responses, resulting in either worsened or improved infection. It is reasonable to consider this, as the immunosuppressive role of GXM and the immunoprotected role of GXMGal have been previously described [24]. Therefore, we investigated some parameters associated with pro-inflammatory and anti-inflammatory response profiles to better understand how these polysaccharides might be contributing to the different infection profiles.

The production of the pro-inflammatory cytokine TNF-α by macrophages plays an important role in the control of *Leishmania* infection [7]. Studies using C57Bl/6 mice, which are normally resistant to leishmaniasis, become susceptible to the disease when deficient in soluble TNF, even developing a Th1 response, presenting a high parasite load, and persistent lesions [59,60,61,62]. As mentioned earlier, GXMGal has been associated with the induction of a pro-inflammatory response, including TNF-α production [63]. In agreement, we demonstrate that this property of GXMGal remains even in macrophages infected with *L. major*. Notably, the increase in TNF-α levels was inversely proportional to the number of parasites, further highlighting the cytokines in controlling the parasite load.

Unlike GXMGal, our results demonstrate that GXM induces the production of IL-10 in macrophages, regardless of *L. major* infection status. Importantly, IL-10 neutralization restores macrophages’ ability to control parasite burden. The role of IL-10 as an immunosuppressive cytokine associated with impaired control and clearance of *Leishmania* has been described in the literature [7,12,64]. The increased production of IL-10 and concomitant low production of TNF-α in GXM-treated cells can be explained by the fact that IL-10 is a potent inhibitor of TNF-α [65,66]. Additionally, we observed that the immunosuppressive property of GXM was maintained in the presence of a parasitic infection.

One of the main mechanisms of *Leishmania* elimination occurs through the production of NO by the enzyme iNOS [67,68]. Mice that are normally resistant to leishmaniasis become susceptible to *L. majo* infection even with a Th1-type response when deficient in iNOS [67,68]. NO production in macrophages can be induced by the stimulation of IFN-γ and/or TNF-α [69,70]. In our study, we observed a significant increase in NO production in peritoneal macrophages infected with *L. major* and treated with GXMGal, but not in those groups treated with GXM. The use of the iNOS inhibitor, L-NIL, significantly reduced NO levels, showing that NO production is indeed induced by GXMGal. We hypothesize that the lack of NO production in infected macrophages treated with GXM is a result of increased IL-10 levels, as this cytokine is a potent inhibitor of NO production [65].

Another important factor associated with parasite persistence is the presence of high levels of PGE_2_ [18,42,71,72,73]. In our experiments, we observed that treatment with both GXM and GXMGal leads to elevated PGE_2_ production. Although PGE_2_ has been associated with infection progression [18,72,73], it has been demonstrated that its effect depends on the microenvironment, where PGE_2_ can exert both pro-inflammatory and immunosuppressive functions [74].

Fungal polysaccharides from *Candida albicans*, such as mannans and β-glucans, are known to induce PGE_2_ production in human cells [75]. However, the role of capsule polysaccharides from *C. neoformans* in the induction of PGE_2_ synthesis has not been described until now. In this study, we demonstrated that both GXM and GXMGal stimulate PGE_2_ synthesis in uninfected murine macrophages. Previous studies have shown that PGE_2_ synthesis primarily occurs through the activity of COX-2 [15,76,77,78]. To better understand the impact of PGE_2_ production in macrophages during *L. major* infection treated with GXM or GXMGal, we used a COX inhibitor that targets both constitutive and inducible forms, aspirin (AAS) [43], and an inducible COX-2 inhibitor, NS-398 [44]. We observed a significant reduction in parasite load in macrophages treated with GXMGal. Interestingly, in infected macrophages treated with GXMGal, inhibition of COX led to an increase in parasitic load, indicating that PGE_2_ synthesis could contribute to the persistence of *Leishmania*. Despite this, Rieser and colleagues had previously demonstrated the pro-inflammatory role of PGE_2_, which, when combined with TNF-α, could induce high levels of IL-12 in human dendritic cells [79]. Similarly, Panaro and colleagues showed that human macrophages stimulated with LPS and PGE_2_ increased their leishmanicidal capacity through enhanced NO production [80]. França-Costa and colleagues detected higher levels of PGE_2_ and TNF-α in samples from patients with cutaneous leishmaniasis, while in patients with diffuse cutaneous leishmaniasis, elevated levels of PGE_2_ and TGF-β were found, demonstrating that depending on the profile of mediators present in the microenvironment, PGE_2_ can exert either pro-inflammatory or anti-inflammatory effects [20]. Therefore, we suggest that a similar mechanism may be occurring in our experiment.

The precise role of PGE2 in our model remains unclear. Although our findings suggest a modulatory effect, they do not definitively clarify whether PGEE2 acts as a protective or detrimental factor during infection. We hypothesize that a feedback effect between PGE2 and cytokines may be involved, whereby PGE2 inhibition could interfere with the synthesis of certain cytokines that interfere with the establishment of infection. However, further experiments are needed to confirm this hypothesis.

Increased PGE_2_ levels led us to investigate the presence of lipid bodies (LBs) following treatment with GXM and GXMGal, as these organelles are known sites for PGE_2_ synthesis during parasitic infections [45,81]. Elevated numbers of LBs have been identified in *T. cruzi* infections [81,82,83,84], and it has also been demonstrated that inhibition of LB biogenesis impairs PGE_2_ synthesis [84]. In leishmaniasis, increased LB formation has been identified in bone marrow-derived macrophages infected with *L. major* [78] and in murine macrophages infected with *L. infantum* [85], with LB accumulation associated with parasitic persistence. It has also been shown that *Leishmania* itself can induce LB formation in macrophages [78]. In our experiments, we observed that macrophages infected with *L. major* displayed elevated LBs levels, demonstrating that the infection alone is sufficient to induce their biogenesis. However, when these cells were treated with GXM or GXMGal, we observed a further increase in cytoplasmic LBs. These polysaccharides are certainly contributing to the increased formation of LBs, as uninfected macrophages treated with GXM or GXMGal also showed significantly elevated LB levels. Previous studies have shown that purified polysaccharide fractions from *Histoplasma capsulatum*, including β-glucan, chitin, galactomannan, and α-glucan, can induce increased LB formation in macrophages [86]. Despite the limited number of studies, this observation, along with our results, indicates that fungal polysaccharide stimulation is sufficient to mobilize LB formation in macrophages. Therefore, we infer that the increased LB formation induced by GXM contributes to the exacerbation of *L. major* infection, as these organelles serve as a nutrient reservoir for the parasite, thereby aiding its intracellular survival [15,42,45,87,88,89]. Conversely, the accumulation of LBs induced by GXMGal, although elevated, seems not to affect the macrophage’s ability to control the parasitic load, primarily due to the pro-inflammatory response of these cells.

Considering the data together, we conclude that GXMGal stimulates a pro-inflammatory macrophage response, characterized by TNF-α and nitric oxide production. Additionally, GXMGal directly contributes to the control of *L. major* proliferation in infected macrophages, by enhancing microbicidal mechanisms that inhibit the intracellular parasite growth. In contrast, GXM induces an anti-inflammatory response in macrophages, with increased production of IL-10, PGE_2_, and TGF-β, and increased LB formation, thereby contributing to the worsening of *L. major* infection.

The findings of this study indicate that GXM and GXMGal exhibit contrasting immunomodulatory roles in the regulation of *Leishmania* infection in murine macrophages. GXM negatively modulates microbicidal mechanisms during in vitro infection of murine macrophages with *L. major*, leading to higher parasite loads after 3 days post-infection, along with lower levels of NO (indicated by nitrite) and TNF-α. In contrast, GXMGal appears to control the parasite load, maintaining low numbers of *L. major,* as observed in macrophages stimulated with IFN-γ alone. Although no differences in parasite burden were observed between GXMGal-treated macrophages and those untreated or treated with IFN-γ, the elevated levels of nitrite and TNF-α in the GXMGal group suggest an enhanced anti-parasitic state of the macrophage activation. Interestingly, GXMGal also stimulates the production of high levels of PGE2 by murine macrophages, even in the absence of *L. major*. This PGE2 induction may contribute to the partially reduced number of intracellular amastigotes observed in GXMGal-treated macrophages, compared to untreated and IFN-treated macrophages. This observation highlights the potential of GXMGal to enhance the host’s antiparasitic response, possibly contributing to infection resolution. Altogether, these results offer new insights into the biological activity of fungal-derived polysaccharides in modulating innate immune responses. Further studies, including in vivo infection models and investigations across different *Leishmania* species, are necessary to validate and expand the understanding of the immunomodulatory properties of GXMGal in parasitic diseases.

Certain methodological considerations, including the use of a single in vitro model, may influence the robustness and generalizability of the conclusions. To address this, future studies will aim to investigate the modulatory effects of capsular polysaccharides in an in vivo infection model and to assess their potential application as vaccine adjuvants. Despite these limitations, the present work offers relevant advances to the field. Previous studies have documented the activity of fungal components against tumor cells and/or microorganisms [90,91]. In this context, our findings contribute to expanding the current body of knowledge and provide valuable insights to guide more comprehensive future investigations.

## Data Availability

The original contributions presented in this study are included in the article. Further inquiries can be directed to the corresponding authors.
